# To Kill, Stay or Flee: The Effects of Lions and Landscape Factors on Habitat and Kill Site Selection of Cheetahs in South Africa

**DOI:** 10.1371/journal.pone.0117743

**Published:** 2015-02-18

**Authors:** Susana Rostro-García, Jan F. Kamler, Luke T. B. Hunter

**Affiliations:** 1 Wildlife Conservation Research Unit, Department of Zoology, University of Oxford, The Recanati-Kaplan Centre, Tubney House, Abingdon Road, Tubney, Abingdon, United Kingdom; 2 Panthera, 8 West 40^th^ Street, 18^th^ Floor, New York, New York, United States of America; University of Tasmania, AUSTRALIA

## Abstract

Understanding how animals utilize available space is important for their conservation, as it provides insight into the ecological needs of the species, including those related to habitat, prey and inter and intraspecific interactions. We used 28 months of radio telemetry data and information from 200 kill locations to assess habitat selection at the 3^rd^ order (selection of habitats within home ranges) and 4^th^ order (selection of kill sites within the habitats used) of a reintroduced population of cheetahs *Acinonyx jubatus* in Phinda Private Game Reserve, South Africa. Along with landscape characteristics, we investigated if lion *Panthera leo* presence affected habitat selection of cheetahs. Our results indicated that cheetah habitat selection was driven by a trade-off between resource acquisition and lion avoidance, and the balance of this trade-off varied with scale: more open habitats with high prey densities were positively selected within home ranges, whereas more closed habitats with low prey densities were positively selected for kill sites. We also showed that habitat selection, feeding ecology, and avoidance of lions differed depending on the sex and reproductive status of cheetahs. The results highlight the importance of scale when investigating a species’ habitat selection. We conclude that the adaptability of cheetahs, together with the habitat heterogeneity found within Phinda, explained their success in this small fenced reserve. The results provide information for the conservation and management of this threatened species, especially with regards to reintroduction efforts in South Africa.

## Introduction

An animal’s habitat selection can be viewed as a hierarchical order of selection processes [1], that is influenced by scale [2–5], and driven by distinct determinants at different spatial scales [6,7]. The hierarchical selection occurs at the physical or geographical range (1^st^ order), in which, from an available area, an individual’s or social group’s home range is selected (2^nd^ order; [1]). Within the home range there is differential use of the various habitat components (3^rd^ order) which could determine the procurement of food items and lead to the selection of feeding sites (4^th^ order; [1]). The habitat selection within home ranges can be influenced by several factors, including resource availability (e.g. [8]), landscape attributes (e.g. [9]), suitability for protection (e.g. [10]), spatiotemporal variations (e.g. [11]) and intra- and interspecific interactions (e.g. [2]).

Among carnivores, interspecific interactions can influence population dynamics [12] and have significant implications for the function and structure of carnivore communities [2,13]. Such interactions often occur as interference competition, manifested as harassment, kleptoparasitism and in some cases intraguild predation [14,15]. This may result in spatial or temporal avoidance, population reduction or exclusion of the subordinate carnivores ([4]; e.g. cape foxes *Vulpes chama* [16]; coyotes *Canis latrans* [14]; African wild dogs *Lycaon pictus* [17]; and cheetahs *Acinonyx jubatus* [18]).

Cheetahs have been reported to suffer from intraguild competition by lions *Panthera leo*, spotted hyenas *Crocuta crocuta* and occasionally leopards *Panthera pardus* [12,19]. These larger predators represent a threat to the smaller-bodied cheetahs [20,21] as they can affect their food intake by limiting access to high resource areas [20] or kleptoparasitism (e.g. 10–12% of kills are kleptoparasitized in Serengeti National Park (SNP); [18]), and reduce population sizes via increased cub mortality (e.g. 73% of cub mortality was due to predation in the SNP; [22]). In response, cheetahs often demonstrate avoidance behavior to minimize interactions with dominant carnivores [23], with spatial and temporal partitioning regarded as the principal behavioral mechanisms by which this is achieved [20,24]. Accordingly, cheetahs have been described as a refugial species that seeks competition refuges within the landscape with low densities of lions and spotted hyenas [20,25].

Due to the cheetah’s high-speed hunting strategy, it was long assumed that the species relied on open habitats such as grasslands [26]. However, research showed that woodland vegetation, often assumed to be sub-optimal for cheetahs, increased cub survival, and therefore was suggested to be key in the ultimate survival of the species [27]. Although cheetahs preferred open over closed habitats in previous studies, they used denser habitat for hunting or to reduce kleptoparasitism [28,29]. In general, females used denser vegetation types than males as they tend to avoid larger carnivores more than males [28,30]. These intersexual differences in habitat use perhaps stem from the cheetah’s sexual dimorphism and the fact that males can form coalitions, whereas females, either solitary or accompanied by dependent cubs, are potentially more vulnerable [21].

A fine-scale understanding of the cheetah’s preference for and success in different habitat types is particularly relevant to the species’ conservation in South Africa where their reintroductions have proliferated in the last two decades. Such reintroductions occur into fenced reserves, many of which are small and had little or no pre-release assessment of suitable habitat [23,31]. The relatively small size of these reserves (10–1,000 km^2^) might reduce access to competition refuges for cheetahs and thus increase the competition between them and other dominant carnivores like lions, which are typically reintroduced simultaneously. Understanding how cheetahs utilize available space with such constraints is important to improve the success of reintroduction efforts. Moreover, given Africa’s rapidly expanding human population and the increasing pressure on the remaining natural strongholds of large carnivores, such areas may become increasingly important for carnivore conservation.

The aim of this study was to assess the habitat selection and hence adaptation of reintroduced cheetahs in the small (170 km^2^) *Acacia*-dominated Phinda Private Game Reserve (hereafter Phinda) in South Africa. Specifically, we examined the potential determinants of 3^rd^ and 4^th^-order habitat selection of cheetahs. Our hypotheses were that:

1. Cheetahs exhibit habitat selection that is driven by different determinants at the different scales.
2. Cheetah habitat selection varies depending on the characteristics of the landscape and levels of lion risk, but in general open habitats and high prey areas are positively selected.
3. Habitat selection, feeding ecology, and avoidance of lions differ by sex and reproductive status of cheetahs.

To test these hypotheses we used data from radio-collared cheetahs and lions, information from cheetahs kill sites, and landscape data from Phinda.

## Materials and Methods

### Study area

**Fig 1:**
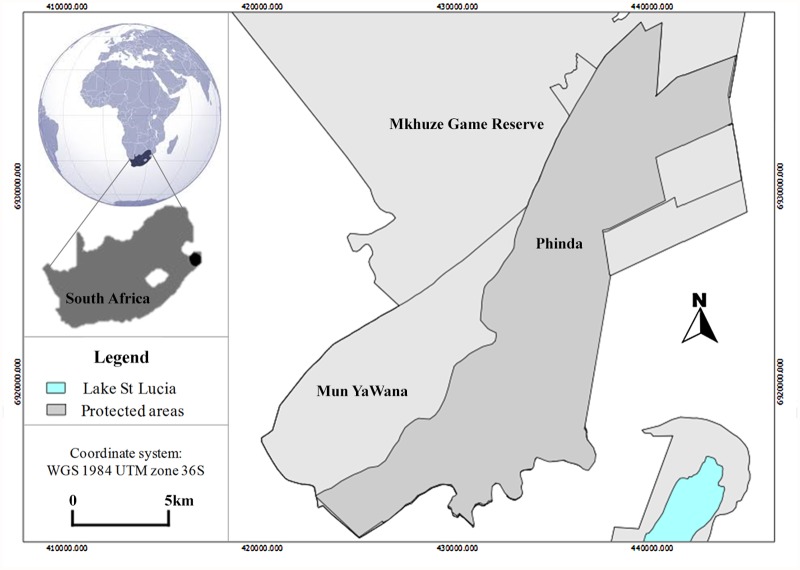
Map of Phinda Private Game Reserve during the study period (1993–1995). The MunYaWana area indicated in the map was consolidated with Phinda in July 2004, forming the MunYaWana conservancy. Light grey polygons show state-run protected areas. The white background is a mosaic of agricultural and communal lands and the light blue polygon shows the Lake St-Lucia. The inset on the left shows the location of Phinda in South Africa.

The study was conducted in the Phinda Private Game Reserve (27˚ 44ˈ–27˚ 55ˈ S, 31˚ 12ˈ–32˚ 26ˈ E), located in the southern Maputaland region of northern KwaZulu-Natal, South Africa (Fig. 1). Phinda is privately owned and was established as a conservation area in 1990. It borders the Mkhuze Game Reserve to the northwest and a matrix of private game reserves, local communities and cattle ranches to the southwest and east. During the 1990s Phinda comprised an area of 170 km^2^ [32], but increased gradually to its current size of 220 km^2^ [33]. It is now part of the MunYaWana conservancy.

The area is predominantly flat with over 95% of the reserve lying beneath 100 meters above sea level (m.a.s.l.) and with an altitude gradient ranging from 4 to 201 m.a.s.l. [32]. The reserve is crossed by two naturally seasonal rivers, the Munyawana that bisects the area at approximately the mid-point between southern and northern tips, and the Mzinene that forms the southern boundary of the reserve. An artificial weir ensured year-round water for the latter. Throughout the reserve, numerous small, shallow, seasonal pans can be found, as well as man-made dams that provide water during the winter on an ad-hoc basis. The climate is warm to hot, humid subtropical [34] with two distinct seasons: a hot, humid summer from October to March, which includes the rainy season, and a warm arid winter from April to September. The area receives an average rainfall of 550 mm, and during the study period ca. 85–90% of it occurred in the rainy season. Mean monthly temperatures varies from 19 ˚C in July to 33 ˚C in January [33].

The reserve is situated in Natal lowveld bushveld, coastal and bushveld-grassland vegetation zones [35]. The prevailing habitat type is broad-leafed woodland [33], dominated by *Acacia* and *Terminalia* spp., interspersed with fire-maintained grasslands and wooded grasslands resultant from previously cultivated areas [36]. Hunter [32] classified nine distinct vegetation types in the reserve: open red sand bushveld, closed red sand bushveld, palmveld, grassland, sandforest, open mixed bushveld, closed mixed bushveld, dry mountain bushveld and riparian woodland (see descriptions in Table 1). A GIS map of these vegetation types was developed from aerial photographs (1:30,000) and confirmed through extensive ground truthing by Hunter [32].

**Table 1:** Summary of the nine main habitat types found in Phinda Private Game Reserve.

Vegetation structure	Habitat type	Preferred prey density^†^	Description	Area (%)
Closed	Closed red sand bushveld (CRS)	Low	Mixed-species woodland with trees 6–10 m tall and scattered thickets, where approximated distance between neighboring trees > 6 m tall was < 10 m. Common tree species are *Acacia burkeii*, *Combetum molle*, *Sclerocarya caffra*, *Ziziphus mucronata*, *Albizia versicolor*, and *Terminalia sericea*	27.4
	Closed mixed bushveld (CMB)	Low	*Acacia*-dominated woodland with poor grass understory, where approximated distance between neighboring trees > 6 m tall was < 10 m. Common tree species include *A*. *tortillis*, *A*. *nilotica*, *A*. *grandicornuta*, *A*. *Senegal*, *Spirostachys Africana*, and *Schotia brachypetala*	20.7
	Sandforest (SF)	Low	Very dense forest with high canopy extending to 25 m, dominated by *Newtonia hilderbrandtii*, *Cleistanthus schlerteri*, *Hymanocardia ulmoides*, *Pteleopsis myrtifolia*, *Dialium schlerteri*, *Croton gratissimus* and *Strychnos henningsii*. *A*lmost no herbaceous understory, but there is a well developed sub-canopy of small trees/shrubs dominated by *Salacia leptoclada*, *Croton pseudopluchellus* and *Hymenocardia ulmoides*	7.1
	Riparian woodland (R)	Medium	Well developed woodland occurring adjacent to the two main rivers, with the main woody species being *Acacia xanthophloea*, *A*. *robusta*, *Spriostachys africana*, *Rauvolfia caffra* and *Trichilia emeti*. A dense subcanopy of trees is dominated by *A*. *schweinfurthii*, *Azima tetracantha* and usually the exotic *Eupatorium odoratum*	3.9
Semi-open	Dry mountain bushveld (DMB)	Medium	Open woodland associated with rocky soils usually > 100 m.a.s.l. The representative species include *Combretum apiculatam*, *Acacia nigriscens*, *Themeda triandra*, *Heterropogon contortus* and *Cymbopogon excavatus*. The herbaceous understory is generally poor, especially where the soil is shallow	6.1
	Open mixed bushveld (OMB)	High	*Acacia*-dominated woodland with poor grass understory, where approximated distance between neighboring trees >6 m tall was > 10 m. Common tree species include *A*. *tortillis*, *A*. *nilotica*, *A*. *grandicornuta*, *A*. *Senegal*, *Spirostachys Africana*, and *Schotia brachypetala*	9.8
	Open red sand bushveld (ORS)	High	Mixed-species woodland with trees 6–10 m tall and scattered thickets, where approximated distance between neighbouring trees > 6 m tall was > 10 m. Common tree species are *Acacia burkeii*, *Combetum molle*, *Sclerocarya caffra*, *Ziziphus mucronata*, *Albizia versicolor*, and *Terminalia sericea*	8.7
Open	Palmveld (P)	Medium	Scattered *Hyphaene natalensis* within an open diverse grassland dominated by *Themedea*, *Eragrostis*, *Aristida*, and *Perotis*. Associated trees include *Phoenix reclinata*, *Dichrostachys cinera*, and *Strychnos madagascariensis*	7.7
	Grassland (G)	High	Tall tussocked grasslands occurring on seasonally inundated floodplains and artificially cleared areas that were formally agricultural fields. Dominant grass species are *Phragmites australis*, *Echinochloa pyramidalis*, *Erichloa spp*. and *Sorghum spp*. on the floodplain, and *Aristida spp*, *Themeda spp*. *Tristachya ssp* and *Paspalum ssp*. on the cleared areas	8.6

^†^ Based on densities of the 2 species (impala and reedbuck) preferred by cheetahs.

Forty-two large mammal species were present in Phinda during the study [9]. Lions and cheetahs were reintroduced in 1992 after anthropogenic extirpations in the region in 1938 and 1941 respectively [32,36]. Spotted hyenas and leopards had persisted in the area, but numbers of both were low in Phinda during the study period due to historical persecution [9,32]. African wild dogs were not resident on Phinda during the study, although transient animals occasionally passed through [32]. As required by the local conservation authority (Ezemvelo KwaZulu-Natal Wildlife), the entire perimeter of the reserve (115 km) was surrounded by a 1.8 m tall electric game fence.

### Felid data collection

For this study we used data from a larger 40-month investigation on the ecology of reintroduced lions and cheetahs on Phinda, conducted from 1992 to 1995 [32]. Between March 1992 and June 1994, 15 cheetahs and 13 lions were released in groups within Phinda on seven separate occasions. Details of the release procedures and monitoring methods are given by Hunter [32]. For each re-introduced group VHF radio-collars (Telonics, Arizona, USA) were fitted to certain individuals before release. After a post-release settling-period, non-essential collars were removed to reduce the aesthetic impact on the tourism experience and only selected individuals that represented each pride or coalition remained collared [32]. Phinda is private property, for which no government permission was required for field anesthesia of felids during the time of the study. Field anesthesia was undertaken by Phinda management personnel as permitted by South African law [32]. In South Africa, the cheetah is a non-endangered species, classified as Vulnerable [32].

For the analysis, we used data from April 1993 to August 1995, as by that time all lions had been released, and their numbers and home ranges were stable. In addition, an internal fence that once divided the reserve in two had been removed. During this period, the telemetry locations of six adult cheetahs and four adult lions (representing three distinct social groups) were monitored. The social groups of cheetahs included two male coalitions consisting of two individuals each, one female with cubs, one solitary female, one female that started as a solitary animal but later had cubs and another female that had cubs and later became solitary. The lion social groups included two female prides and one male coalition. During the study period additional cheetahs were present in the reserve but were not included in the analysis due to insufficient data. For example, a third cheetah male coalition was released during the last reintroduction event (i.e. 27 months from the start of the study), thus was not monitored long enough to obtain sufficient data for the analysis. Additionally, grown offspring of collared individuals, if not translocated, became independent at different times during the study period. The exclusion from the analysis of the above animals should not affect our results as the majority of the cheetah social groups were monitored at any given time, thus sufficiently representing the species’ use of habitat. Similarly, collared cheetahs, all of which were adult residents, used the entire reserve, thus it was assumed that non-collared cheetahs had minimal influence on the space use of collared individuals. Importantly, all adult lion groups were monitored during the study period, thus lion risk to cheetahs could be accurately assessed. The other dominant carnivores of the area (i.e. spotted hyenas and leopards) were unlikely to represent a significant risk to cheetahs given their low numbers on Phinda during the study period.

Two types of data collected from cheetahs were used in the analysis: location data, and kill site data. Location data came from daily attempts to locate collared individuals by using a vehicle to home in on radio signal until felids were sighted, then the location was recorded with a GPS unit. All felids, including the offspring, were recognized individually by unique pelage markings and whisker spots. We used a maximum of one location per day per animal, hence locations were considered independent, and temporal correlation, if existent, should have been limited. Kill sites were located using three different methods which included direct observations, radio-location observations, and ranger reports confirmed by Hunter [32]. Direct observations were made when following radio-collared individuals that made kills. To adequately sample kills from all collared animals, on a rotational basis individuals were continuously followed for a complete day until they made a kill. Therefore, the data were not likely to be biased towards large prey. Radio-location observations occurred when located felids had already made a kill. In these cases, it was recorded whether the prey was killed or scavenged by the felid(s) feeding on it, or had an unknown origin [32]. Ranger reports came from direct observation of rangers during game drives, and the information was recorded and, when possible, confirmed by Hunter [32]. For all kills, the prey species, prey age (i.e. juvenile, sub-adult, adult), coordinates (obtained from a handheld GPS unit), and habitat type were recorded, and when possible, the time of kill and sex of the prey. The prey weight of all kills was estimated based on published data of herbivore sex-age classes [32]. Initial analysis using chi-square tests showed there was no significant difference in prey size recorded based on the three methods of collecting kill data [32], thus kill data were pooled for subsequent analyses.

To assess whether cheetahs used prey or habitat types in accordance to their availability, we used Jacobs’ index *D* [37]: *D* = *r* - *p*/*r* + *p* - 2*rp*) where *r* is the fraction of habitat or prey used, and *p* is the fraction of habitat or prey available. The index ranges between −1 (maximum avoidance) and +1 (maximum preference). Jacobs’ indices were calculated to investigate prey selection using prey population estimates from Hunter [32] and to examine habitat selection at scales not included in the models (i.e. home ranges vs. study areas, kill sites vs. home ranges). For analysis of prey selection, because *D*-values of rare species often are biased [38], we excluded species that were < 3% used and < 3% available, thus giraffes *Giraffa camelopardalis* were not considered in the analysis.

Previous research reported sex and group size differences in cheetah behavior (e.g. [23]). Therefore, we used chi-square tests to compare cheetah social groups with regards to habitat of kill sites, and the size, age, and sex of the prey killed. We also used general linear models to compare the level of lion encounter risk at kill sites among the different habitat types and cheetah social groups.

### Landscape data

Landscape features were expected to be relevant for our analysis as previous research showed differential use of available areas by cheetahs [39]. Therefore, we developed a geographic information system (GIS) to depict the landscape attributes of the study area. We used the GIS map of Phinda’s nine vegetation types, and to disentangle the role of habitat characteristics and prey, we classified the habitats as high, medium, and low prey, based on the density of the cheetahs’ preferred prey (Table 1). Elevation was obtained at 30 m resolution from the global digital elevation model of the Advanced Spaceborne Thermal Emission and Reflection Radiometer (ASTER GDEM; http://asterweb.jpl.nasa.gov/gdem.asp).

Additional features that were expected to be determinants of cheetah habitat selection were water sources, roads, and Phinda’s perimeter electrical fence (hereafter boundary). Water sources were assumed to be important not only for direct consumption but also because the distribution of prey is constrained by that of water, particularly during the dry season [40]. However, lions have been shown to select sites close to water holes [41], imposing a potential risk for cheetahs in these places. To disentangle the role of water sources in cheetah habitat selection, GIS layers for water sources were used, including rivers and waterholes (i.e. pumped and natural) found throughout Phinda. Based on patterns observed in the data, we separated for illustrative purposes the effects of water holes into 2 categorical distances: close to water sources (< 300 m), and farther away from water sources (> 300 m). Because roads are frequently used to travel by large carnivores, including cheetahs in thicket-dominated areas [42], we analyzed the role of roads in cheetah habitat selection using a GIS layer of Phinda’s road network. Finally, because physical barriers influence species distribution and ranging behavior [43], we developed a GIS layer of the boundary to investigate if this affected cheetah habitat selection. Raster cost surfaces (30 m resolution, [44]), indicating the distance of each cell to the closest road, water sources and boundary were developed from the GIS layers using the GRASS (v.6.4 [45]) r.cost module. GRASS was used for the spatial integration and development of the layers whereas Quantum GIS (v.1.8.0-Lisboa [46]) was used to visualize and generate maps (projection: UTM; units: meters, datum: WGS 1984, Zone 36 South).

### Lion encounter risk

Using independent telemetry locations (i.e. maximum one location per day) of Phinda’s three lion prides from the study period, a ‘landscape of lion risk’ raster map was developed for the entire reserve based on the summation of each pride’s resistant cumulative kernels, calculated with UNIversal CORridor network simulator (UNICOR v 2.0 [47]; electric fence included as an impermeable barrier to lion movement). Lions form cohesive groups [48] and non-collared lions in the study were almost always associated with collared individuals, with indexes of association ranking from 0.73 to 0.91 [36]. Thus, data from radio-collared individuals were assumed to represent movements of the lion group in which the collar was deployed. The resulting map represented the probability of lion presence over the long-term (i.e. cumulative lion risk) in an area and was used to investigate the role that lions had on cheetah habitat selection at both the 3^rd^ and 4^th^ order.

### Third-order habitat selection

We investigated if cheetahs selected for specific habitats within their home range (3^rd^-order habitat selection) using location data. We assessed the resource selection of cheetahs in a use-available design [49], where the resource used, estimated from independent cheetah locations, was compared to resources at randomly sampled locations within cheetah home ranges using mixed-effects logistic regression models to develop resource selection functions (RSF; [50]). Cheetah home ranges were based on 90% isopleths [25,51] of resistant cumulative kernels calculated with UNICOR (v 2.0). To characterize availability, random points were generated within cheetah home ranges using the ‘‘Random Points” option of the Vector Research Tools of QGIS. The same number of random points as the number of cheetah locations were generated to achieve a 1:1 ratio of used-to-available [52]. The variables (i.e. landscape and lion encounter risk) values at each used and available point were extracted using the point sampling tool in QGIS. Generalized Linear Mixed-effects Models (GLMM) with a binomial error structure and logit link function were used, with 0/1 as binomial response variables—where 1 represented cheetah locations and 0 the randomly generated points. GLMM RSF models were developed with the lme4 package [53] of R software [54]. In each model, the identity of the cheetah was entered as a random intercept to control for the lack of independence of points within individuals and unbalanced sample sizes [55]. Explanatory variables were habitat type, cheetah sex, lion risk, elevation, and distance to water, boundary and roads. The interaction between landscape variables and lion risk was evaluated as habitat use of lions also was assumed to be affected by these factors. A 2^nd^-order interaction between habitat type and distance to water was also included. All the continuous variables were scaled using R’s scale command. Given previously reported sex differences in cheetah ecology (e.g. [23]), we also ran 3^rd^-order GLMM separately for females and males.

### Fourth-order habitat selection

We investigated whether cheetahs selectively killed in certain habitats (4^th^-order habitat selection) using kill site data. This was done initially by comparing cheetah kill site characteristics to random non-kill locations selected from cheetah locations in a 1:1 ratio approach. However, because results differed among analyses of four different random samples, we compared cheetah kill site characteristics to all cheetah non-kill locations. This was expected to better represent the mean of the sample, while also allowed to use all the available data. GLMM with a binomial error structure and logit link function were used, with 0/1 as binomial response variables—where 1 represented cheetah kill sites and 0 cheetah non-kill locations. Logistic regression was used to model the probability of a site being a kill site as a function of habitat type, season, distance to water, boundary, and roads, as well as lion risk, and the interaction of the latter with landscape variables. In addition, the cheetah group identity (i.e. male coalition, solitary female, female with cubs) was used as a fixed factor, and the interaction between lion risk and cheetah group identity investigated. In each model, the identity of the cheetah was entered as a random intercept [55]. A 2^nd^-order interaction between habitat type and distance to water also was preliminarily included in the model. Nonetheless, due to memory size limitations, this interaction could not be evaluated for the entire data set. Given that the results from the above analysis showed seasonal differences, we ran a second set of analyses per season.

All statistical analyses were performed using statistical software R [54] and significance was considered when *P* < 0.05, whereas marginal significance was considered when *P* ranged from 0.05 to 0.10. In order to exclude highly correlated variables (r > 0.70 or r < -0.70) from the models we tested for possible correlations (using Pearson’s correlation) but none were found. The multi-model inference R package MuMin [56] was used to run all possible models and to select models using the Akaike Information Criterion corrected for small samples (AIC_c_). Given that none of the considered models achieved a high AIC_c_ weight (i.e. > 0.9) model averaging with MuMin [57] was done to calculate the average beta coefficient and relative importance of the predictors. Finally, we plotted the magnitude of effects for the most important continuous parameter estimates, which were based on both statistical and biological significance, and calculated the odds ratios (OR) and confidence intervals (CI) for the most important categorical parameter estimates. The plots are the result of generalized linear models with a binomial error structure and represent a model of the relationship between the response variable (i.e. probability of cheetah presence) and a single predictor variable (without the other predictor variables included).

## Results

### Home range characteristics and 3^rd^-order habitat selection

A total of 1,669 cheetah locations were used for the analyses, with an average of 278 locations per cheetah social group (range 90–630, *n* = 6). The cheetah home ranges included more grassland (*D* = +0.24), open mixed bushveld (*D* = +0.15) and palmveld (*D* = +0.13) than would be expected given the overall availability within the study area (2^nd^-order), and considerably less dry mountain bushveld (*D* = -0.50) and sandforest (*D* = -0.38). Mean home-range size was 22.04 km^2^ (SE = 8.36 km^2^) for male coalitions (*n* = 2) and 30.07 km^2^ (SE = 11.44 km^2^) for females (*n* = 4).

Based on data pooled across sexes and seasons, the 3^rd^-order selection (locations vs. random points within home ranges) showed that cheetahs varied their use of habitats in relation to several parameters (Table 2). Cheetahs positively selected open mixed bushveld (OR 1.38, CI: 1.07–1.78), palmveld (OR 1.92, CI: 1.25–2.97) and grassland (OR 2.06, CI: 1.62–2.61) areas, with the last two being the most highly selected. In contrast, cheetahs negatively selected places with open red sand bushveld (OR 0.68, CI: 0.49–0.96).

**Table 2:** Third-order habitat selection (locations vs. randomly sampled locations) of cheetahs (*n* = 6), showing multi-model (Generalized Linear Mixed Models) beta coefficient averages of parameters (within the intercept are included closed mixed bushveld, female cheetahs and random locations).

Parameter	Estimate^+^	Std. Error	z value	Pr(>|z|)		Relative importance ^†^
(Intercept)	-0.168371	0.087248	1.93	0.0536	*	
Closed Red Sand Bushveld (CRS)	-0.122657	0.139218	0.881	0.3783		1
Dry Mountain Bushveld (DM)	-0.007216	0.424413	0.017	0.9864		1
Grassland (G)	0.72223	0.118998	6.069	< 2e-16	***	1
Open Mixed Bushveld (OMB)	0.322033	0.126121	2.553	0.0107	*	1
Open Red Sand Bushveld (ORS)	-0.38	0.171626	2.214	0.0268	*	1
Palmveld (P)	0.654387	0.217507	3.009	0.0026	**	1
Riparian woodland (R)	0.46583	0.271512	1.716	0.0862	.	1
Sand Forest (SF)	0.122802	0.275725	0.445	0.6561		1
Boundary (B)	-0.686139	0.086069	7.972	< 2e-16	***	1
Elevation (E)	0.266828	0.108687	2.455	0.0141	*	0.93
Lion risk (LR)	0.039006	0.192945	0.202	0.8398		1
Water bodies (WB)	0.272738	0.224446	1.215	0.2243		1
CRS x LR	-0.041188	0.268182	0.154	0.8779		0.86
DM x LR	-2.78048	0.916512	3.034	0.0024	**	0.86
G x LR	-0.130612	0.278386	0.469	0.6389		0.86
OMB x LR	-0.502081	0.318032	1.579	0.1144		0.86
ORS x LR	-0.462896	0.301319	1.536	0.1245		0.86
P x LR	0.356258	0.558891	0.637	0.5238		0.86
R x LR	-0.204515	0.434912	0.47	0.6382		0.86
SF x LR	0.280031	0.403715	0.694	0.4879		0.86
CRS x WB	-0.612924	0.284168	2.157	0.031	*	1
DM x WB	-0.915662	0.800278	1.144	0.2526		1
G x WB	0.425943	0.283966	1.5	0.1336		1
OMB x WB	-0.63675	0.320913	1.984	0.0472	*	1
ORS x WB	0.400361	0.309937	1.292	0.1964		1
P x WB	0.227858	0.411857	0.553	0.5801		1
R x WB	-0.368235	0.486078	0.758	0.4487		1
SF x WB	-1.304445	0.807229	1.616	0.1061		1
LR x E	0.335293	0.207579	1.615	0.1063		0.54
LR x WB	-0.387709	0.161437	2.402	0.0163	*	0.86
LR x B	0.278652	0.229191	1.216	0.2241		0.44
Males	-0.080313	0.082257	0.976	0.3289		0.37
Roads (Ro)	0.067413	0.089826	0.75	0.453		0.41
LR x Ro	0.121637	0.187783	0.648	0.5171		0.13

‘.’ *P* < 0.1

‘*’ *P* < 0.05

‘**’ *P* < 0.01

‘***’ for *P* < 0.001.

^+^ Effect sizes have been scaled.

^†^ Sum of the *Akaike weights* over all of the models in which the parameter of interest appears.

**Fig 2:**
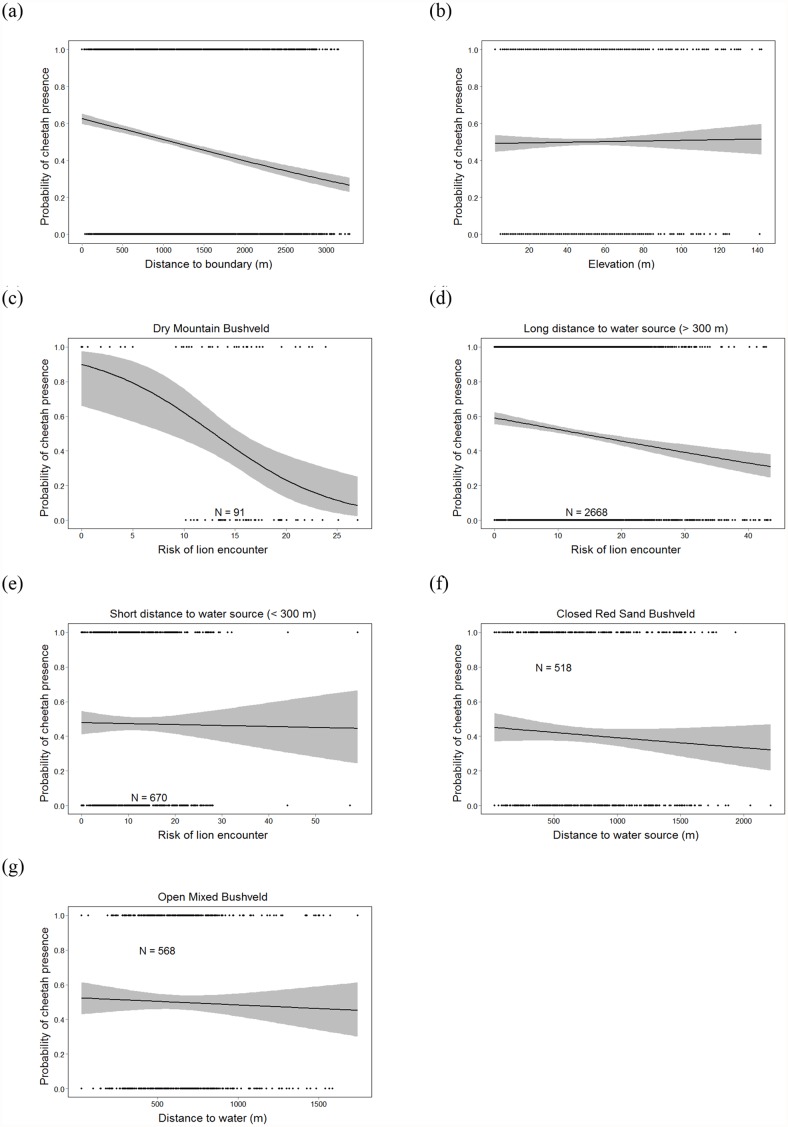
Probability of cheetah presence within their home range in relation to: (a) distance to the boundary; (b) risk of encountering a lion in dry mountain bushveld; risk of encountering a lion at (c) long and (d) short distances to water sources; (e) distance to water sources within closed red sand bushveld. Fitted lines are displayed with 95% confidence intervals.

Areas closer to the boundary also were positively selected by cheetahs (Fig. 2a). Although there was no overall avoidance of areas with high probability of lion presence (i.e. lion risk), such a pattern was observed in dry mountain bushveld (Fig. 2b) and in areas farther away from water (Fig. 2c). Cheetahs used areas near water sources (< 300 m) without any sign of the lion risk avoidance that they showed at greater distances from water (Fig. 2d). In closed red sand bushveld, cheetahs also were less likely to be found in areas farther away from water sources (Fig. 2e).

Female cheetahs, in addition to having the same positive selections reported for the pooled data, selected for places within their home ranges with dry mountain bushveld (OR 3.94, CI: 1.19–13.13) and riparian woodland (OR 2.57, CI: 1.00–6.61; S1 Table). In contrast, male cheetahs selected against places within their home range with dry mountain bushveld (OR 0.27, CI: 0.13–0.55; S2 Table). Females, but not males, selected against places within closed red sand bushveld when farther away from water sources (S1a Fig.). Both females and males negatively selected places farther from the boundary, however for male cheetah coalitions this negative selection also increased as lion risk increased, with a higher likelihood of finding male cheetah coalitions in places closer to the boundary that had low lion risk (S1b Fig.). Male coalitions were less likely to be found where there was a higher chance of encountering lions when they were in dry mountain bushveld, open red sand bushveld or riparian woodland (S1c Fig.). Furthermore, unlike females who showed no selection for areas of different elevation, males positively selected higher areas (S1d Fig.). Finally, there was a trend for male cheetahs to select areas within their home range farther away from roads (S1e Fig.).

### Feeding ecology and 4^th^-order habitat selection

A total of 200 cheetah kills, representing 12 prey species, were recorded during the study period from 6 collared cheetahs $(\bar{X}$ = 33.3 kills per cheetah, median = 17.50). The four most frequently killed species (grey duiker *Sylvicapra grimmia*, impala *Aepyceros melampus*, southern reedbuck *Redunca arundinum* and nyala *Tragelaphus angasii*) comprised 86.5% of the kills (Table 3). The most preferred species was southern reedbuck (*D* = +0.84), followed by impala (*D* = +0.26) and nyala (*D* = +0.03), whereas greater kudu *Tragelaphus strepsiceros*, warthog *Phacochoerus aethiopicus*, blue wildebeest *Connochaetes taurinus* and plains zebra *Equus quagga* were killed less than expected given their availability (all *D* < -0.40).

**Table 3:** Species and age group break down of kills made by cheetahs (*n* = 6) during the 28-month study period (April 1993-August 1995).

Prey species	Kills		Count		
	No.	%	Adult	Juvenile	Sub-adult
Blesbok	1	0.5		1	
Giraffe	2	1.0		2	
Grey duiker	13	6.5	11	2	
Impala	76	38.0	40	22	14
Kudu	3	1.5		3	
Nyala	62	31.0	32	23	7
Red duiker	3	1.5	3		
Southern reedbuck	22	11.0	14	5	3
Steenbok	3	1.5	3		
Warthog	6	3.0		2	4
Wildebeest	4	2.0		2	2
Zebra	5	2.5		4	1

Medium size prey (21–100 kg) accounted for 54.5% of the kills when data were pooled for all cheetahs. However, there was a significant difference in the way cheetah social groups utilized different-sized prey (χ^2^ = 45.58; d.f. 8; *P* < 0.0001; S2a Fig.). For example, small size prey (< 20 kg) comprised 63.2% of the diet of solitary females, compared to only 20.0% and 21.2% for females with cubs and male coalitions, respectively. The diet of male coalitions comprised 24.0% large animals (> 100 kg) whereas females, either solitary or with cubs, did not kill large prey. Concerning the age of prey, adults comprised 51.5% of kills made by all cheetahs, followed by 33.0% juveniles and 15.5% subadults. There was a significant difference among the cheetah social groups and the age of prey killed (χ^2^ = 13.023; d.f. 4; *P* = 0.011; S2b Fig.). Regarding the sex of prey, kills from all cheetahs comprised 38.5% females, 26.5% males, and 35.0% unknown, with a significant difference among cheetah social groups and the sex of prey killed (χ^2^ = 23.153; d.f. 4; *P* < 0.0001; S2c Fig.).

Most cheetah kills occurred in grassland (23.0%), closed mixed bushveld (20.0%) and closed red sand bushveld (18.5%). There was a marginal significant difference (χ^2^ = 23.531; d.f. 16; *P* = 0.100) between the cheetah social group and the habitat type where kills were made. For example, whereas solitary females made 31.6% of the kills in closed mixed bushveld, females with cubs and male coalitions made only 20.9% and 15.2% of their kills in these habitats. In contrast, 10.5% of the kills made by solitary females were in grassland, whereas more kills occurred in this habitat type for females with cubs (21.7%) and male coalitions (28.8%). Within their home ranges, cheetahs (sexes pooled) made more kills than expected in grassland (*D* = +0.29), dry mountain bushveld (*D* = +0.18), and sandforest (*D* = +0.15). The level of lion risk at the location of kill sites (values range: 0.97–59.02) differed with a marginal significance among habitat types (*F*_*8*, *191*_ = 1.983; *P* = 0.051), with kill sites within open red sand bushveld having the highest probability of lion encounter $(\bar{X}$ = 15.76) compared to sandforest, which had the lowest $(\bar{X}$ = 5.99). Moreover, the risk of lion encounter at kill sites differed significantly among cheetah social group (*F*_*2*,*197*_ = 4.369; *P* = 0.014), with male coalitions killing in areas with higher lion risk than solitary females (S3 Fig.).

Of those habitats used within home ranges, cheetahs positively selected for sandforest (OR 3.17, CI: 1.18-.852) and closed red sand bushveld (OR 1.86, CI: 1.08–3.19; Table 4) as kill sites. Moreover, cheetahs had a positive selection for kill sites in closed red sand bushveld as the probability of encountering a lion increased. Among the different social groups, females with cubs were significantly more likely to make kills than solitary females. Overall, cheetahs were significantly less likely to kill prey during winter than summer.

**Table 4:** Fourth-order habitat selection (kill sites vs. locations) of cheetahs (*n* = 6), showing multi-model (Generalized Linear Mixed Models) beta coefficient averages of parameters (within the intercept are included closed mixed bushveld, summer, solitary female cheetahs and random locations).

Parameter	Estimate^+^	Std. Error	z value	Pr(>|z|)		Relative importance^†^
(Intercept)	-2.18273	0.32749	6.665	<2e-16	***	
Winter	-0.3299	0.16825	1.961	0.0499	*	0.71
Closed Red Sand Bushveld (CRS)	0.61812	0.27106	2.28	0.0226	*	0.98
Dry Mountain Bushveld (DM)	0.47585	0.49622	0.959	0.3376		0.98
Grassland (G)	-0.19799	0.24623	0.804	0.4213		0.98
Open Mixed Bushveld (OMB)	-0.29433	0.2657	1.108	0.268		0.98
Open Red Sand Bushveld (ORS)	0.48568	0.3517	1.381	0.1673		0.98
Palmveld (P)	-0.43178	0.35153	1.228	0.2193		0.98
Riparian woodland (R)	0.28629	0.45715	0.626	0.5312		0.98
Sand Forest (SF)	1.15467	0.49392	2.338	0.0194	*	0.98
Water bodies (WB)	-0.24292	0.16508	1.472	0.1411		0.56
Roads (Ro)	0.16767	0.16016	1.047	0.2951		0.49
Boundary (B)	0.17392	0.16563	1.05	0.2937		0.39
Females with cubs (FC)	0.64045	0.28167	2.274	0.023	*	0.37
Male coalitions (MC)	0.30674	0.31402	0.977	0.3287		0.37
Lion risk (LR)	-0.09801	0.39943	0.245	0.8062		0.42
Elevation (E)	-0.05921	0.19407	0.305	0.7603		0.28
LR x Ro	-0.28805	0.36404	0.791	0.4288		0.08
LR x WB	-0.18032	0.2878	0.627	0.531		0.08
FC x LR	0.90802	0.55654	1.632	0.1028		0.07
MC X LR	0.7056	0.57394	1.229	0.2189		0.07
CRS x LR	0.95652	0.49815	1.92	0.0548	.	0.01
DM x LR	0.61099	1.07918	0.566	0.5713		0.01
G x LR	0.56734	0.54394	1.043	0.2969		0.01
OMB x LR	0.76188	0.6046	1.26	0.2076		0.01
ORS x LR	0.86207	0.55887	1.543	0.1229		0.01
P x LR	-0.43768	1.49188	0.293	0.7692		0.01
R x LR	0.55012	0.77111	0.713	0.4756		0.01
SF x LR	-3.60922	3.08346	1.171	0.2418		0.01

‘.’ *P* < 0.1

‘*’ *P* < 0.05

‘**’ *P* < 0.01

‘***’ for *P* < 0.001.

^+^ Effect sizes have been scaled.

^†^ Sum of the *Akaike weights* over all of the models in which the parameter of interest appears.

Concerning the seasonal analyses, during winter cheetahs selected against palmveld (OR 0.30, CI: 0.10–0.90) as kill sites. In contrast, within closed red sand bushveld, cheetahs were marginally more likely to make kills in areas where there was a higher chance of encountering lions (S3 Table). As in the annual model, females with cubs were significantly more likely to kill than solitary females and a similar tendency was evidenced for male coalitions compared to solitary females. There was also a trend (marginally significant) for cheetah kill sites to be farther away from roads.

During summer, cheetahs positively selected closed red sand bushveld (OR 2.50, CI: 1.09–5.75) as kill sites, and marginally selected against open mixed bushveld (OR 0.51, CI: 0.24–1.10; S4 Table). In this season, cheetahs were significantly more likely to make kills farther away from the boundary. Moreover, in this period the probability of having a cheetah kill site decreased as the distance to water increased within grassland, open mixed bushveld and palmveld habitats, with the first two being the most highly significant (S4 Fig.).

## Discussion

Reintroduced cheetahs at Phinda made complex selections regarding habitat use which would not be apparent without fine-scale analysis. Cheetahs positively selected more open habitats with high prey densities at the 3^rd^-order selection (i.e. selection of habitats within home ranges), whereas they positively selected closed habitats with low prey densities at the 4^th^-order selection (kill sites). Such contrasting results show the importance of recognizing the effect of scale in species’ habitat selection, and the need to consider scale when managing habitats to increase suitability for a species [5,41]. Further, our results reinforce that cheetahs are more adaptable than often described and their popular depiction as open plains or grassland specialists is limited [26].

The general pattern of positive selection by cheetahs for open habitats rich in prey was not surprising, and it supported our second hypothesis. Previous studies showed that cheetahs selected for open habitats [26,28,58,59], and selection for areas with high prey abundance has been reported not only for cheetahs [28], but also other felids such as lions [41] and Iberian lynx *Lynx pardinus* [60]). Selection for open habitats by cheetahs also may have allowed them to better detect approaching danger such as lions. The benefits associated with an increased ability to detect predators in open habitats have been suggested for prey species [61–63]. Among carnivores, a similar strategy has been observed in African wild dogs, which, like cheetahs, experience intraguild competition from larger carnivores [64].

Within their home ranges, cheetahs selected against open red sand bushveld, a semi-open habitat with high densities of their preferred prey. Nevertheless, this habitat also contained high densities of the main prey species of lions (i.e. zebra, wildebeest, warthog and nyala; [32]). Therefore, cheetahs likely selected against this habitat to minimize encounters with lions, a strategy used by subordinate species like cheetahs in other areas [27]. Consequently, we conclude that the habitat selection of cheetahs within their home ranges was likely driven by a trade-off between prey abundance and predator avoidance. This also explains why, despite high lion risk, cheetahs used areas near water sources, a feature positively selected by prey [61], whereas they selected against high lion risk areas farther from water, where prey densities were lower. The potential benefits outweigh the risks in the former but not in the latter case. Similarly, cheetahs selected against dry mountain bushveld which has medium densities of their preferred prey, as lion risk increased. This benefit-risk balance also may explain increased cheetah presence closer to the reserve boundary, as lions in Phinda did not favor these areas [32]. Van Dyk and Slotow [65] also showed that high-fenced boundaries are relatively lion-free, thereby allowing subordinate carnivores to have high use of these areas.

At the 4^th^-order selection, cheetahs positively selected kill sites in closed habitats with low densities of their preferred prey, which did not support our second hypothesis. Habitat selection at kill sites may have been driven by two main factors—prey catchability and risk avoidance—similar to the 3^rd^-level selection but with an inverse effect. Prey catchability was likely higher in closed habitats as group sizes of prey species and their ability to detect predators are lower compared to open habitats. Lions and leopards often selected for dense habitats (usually thickets) which increased prey susceptibility, and thus hunting success increased even if these habitats contained lower prey numbers [9,66]. Additionally, it has been suggested that cheetahs use thickets for stalking and ambushing prey, as well as to reduce chase times compared to open habitats [26,59], thus the same was likely true in Phinda. The use of closed habitats for kill sites also likely reduced the risk to cheetahs, their cubs, and of kleptoparasitism, by diminishing detection by lions and other scavengers (e.g. vultures, hyenas) that are attracted to cheetah kills [18]. Retention of kills was longer and kleptoparasitism rates were lower in thicket vegetation for cheetahs in other areas of South Africa [23,26]. In Phinda, cheetahs rarely left kills before they were satiated, and only two carcasses were taken by lions [32], suggesting that kill site selection effectively reduced kleptoparasitism.

The ability of cheetahs to conceal themselves from predators in closed habitat may explain the somewhat unexpected result of increased probability of cheetah kills in high lion risk areas within closed red sand bushveld. Assuming that cheetahs did not actively look for areas of high lion risk, this suggests that within this habitat it was possible that lions could not detect cheetahs effectively. If so, it would seem that cheetahs were responding to the immediate risk of encountering lions (i.e. reactive response), rather than responding to the long-term risk (i.e. predictive response). Thus, if lion presence is spatially detected through immediate signs (e.g. visual, auditory) then the level of risk can be assessed and behavior adjusted accordingly, allowing cheetahs to make and retain kills closer to lions in closed habitat than they would in open habitats. Previous research by Broekhuis et al. [25] in Botswana supports this interpretation, as the spatial distribution of cheetahs appeared to be influenced more by the immediate risks imposed by larger carnivores than by the long-term risk. We believe this strategy was particularly important to cheetahs in Phinda which, being small and fenced, made avoiding larger carnivores especially challenging.

Our results also showed sex differences in habitat selection, feeding ecology, and response to risk by cheetahs, supporting our third hypothesis. Female cheetahs positively selected areas with more closed habitats within their home range compared to male coalitions, similar to that reported in other areas [28,30]. Female cheetahs are smaller and more solitary than males, and often have cubs to protect, making them more vulnerable to larger predators, necessitating a more extensive use of closed habitats [23,28]. This is also supported by observations made by Hunter [32] during the study, where on ten occasions cheetah cubs successfully sought shelter in thick cover when faced with approaching lions. The male coalitions’ selection against high lion risk areas (including habitat with high density of main lion prey) could be due to an active spatial avoidance of lions. Because male coalitions consumed larger prey than females, their diets overlapped more with lions [32], indicating greater potential competition for food resources. Furthermore, two adult male cheetahs might be more visible to lions, particularly when hunting large prey which attracts even more scavengers [12].

At the finest scale (4^th^-order) the results also showed intraspecific differences, but principally between females with and without cubs, similar to that reported for females in the Serengeti [67]. The fact that females with cubs had a greater probability than solitary females of making a kill, and hunted larger prey, seems reasonable due to the higher energetic demands imposed by raising cubs. However, given the relatively low sample size of solitary females in our study, the dynamics between the different female social groups could not be explored in more detail. Thus, we encourage additional research on this topic as it may shed light on more fine-scale differences between cheetah social groups.

## Conclusions

We conclude that habitat selection by cheetahs in Phinda was driven by a trade-off between resource acquisition and lion avoidance, and that the balance of this trade-off varied with scale and cheetah social groups. Our results support earlier suggestions that cheetahs are more of a habitat generalist than previously assumed [23,26]. The adaptability of cheetahs, together with the habitat heterogeneity found within Phinda, may explain their success in this small reserve, evidenced by a high survival rate of cubs and high population growth rate [32]. While our results demonstrate that small fenced reserves dominated by *Acacia* woodlands can support cheetah populations, lion numbers on Phinda were managed below carrying capacity. Moreover, the relatively high numbers of non-migratory prey and the low densities of other competitors (e.g. leopards, hyenas) probably benefitted cheetahs [32]. The ability of cheetahs to successfully find suitable habitat in small reserves as we have demonstrated would be tested if competitors or prey populations occurred at significantly different levels [31]. Thus, if small, fenced reserves are to play a role in cheetah conservation, important site-specific management decisions need to be made with regards to area, prey, and predators numbers, all of which have to be balanced with the touristic functionality of the sites. Finally, although small fenced reserves like Phinda can support apparently self-sustaining populations of cheetahs, they have to be intensively managed and are unlikely to enhance metapopulation persistence [68]. Therefore, effective long-term conservation of cheetahs in the region will be improved immeasurably by removing barriers (mainly fences) and fostering connectivity between existing protected areas, while also providing effective protection for the free-ranging populations of this threatened species.

## Supporting Information

S1 Fig Relationship between the presence of: (a) female cheetahs and the distance to water sources in closed red sand bushveld; (b) cheetah male coalitions and the risk of encountering a lion in close distance to the boundary; (c) cheetah male coalitions and the risk of encountering a lion in different habitat types; (d) cheetah male coalitions and elevation; and (e) cheetah male coalitions and distance to roads. (PDF)

S2 Fig Prey categories by: (a) weight, (b) age, and (c) sex of kills (*n* = 200) made by cheetahs from three types of social groups (F = solitary females, FC = females with cubs, MC = male coalitions). (PDF)

S3 Fig Risk of lion encounter at kill sites among habitat types and cheetah social groups (F = single females, FC = females with cubs, MC = male coalitions). (PDF)

S4 Fig Relationship between distance to water source and the probability of cheetah feeding site within grassland, open mixed bushveld and palmveld habitats during summer. (PDF)

S1 Table Third-order habitat selection (locations vs. randomly sampled locations) of female cheetahs (*n* = 4), showing multi-model (Generalized Linear Mixed Models) beta coefficient averages of parameters (within the intercept are included closed mixed bushveld and random locations). (DOCX)

S2 Table Third-order habitat selection (locations vs. randomly sampled locations) of male cheetah coalitions (*n* = 2), showing multi-model (Generalized Linear Mixed Models) beta coefficient averages of parameters (within the intercept are included closed mixed bushveld and random locations). (DOCX)

S3 Table Fourth-order habitat selection (kill sites vs. locations) of cheetahs (*n* = 6) in winter, showing multi-model (Generalized Linear Mixed Models) beta coefficient averages of parameters (within the intercept are included closed mixed bushveld, solitary female cheetahs and random locations). (DOCX)

S4 Table Fourth-order habitat selection (kill sites vs. locations) of cheetahs (*n* = 6) in summer, showing multi-model (Generalized Linear Mixed Models) beta coefficient averages of parameters (within the intercept are included closed mixed bushveld, solitary female cheetahs and random locations). (DOCX)
