# Supplementary material for: To Kill, Stay or Flee: The Effects of Lions and Landscape Factors on Habitat and Kill Site Selection of Cheetahs in South Africa
Source: PLoS One. 2015 Feb 18;10(2):e0117743. doi: 10.1371/journal.pone.0117743 (PMC4333767; doi:10.1371/journal.pone.0117743)
Supplement: S1 Fig — (PDF) [file pone.0117743.s001.pdf]

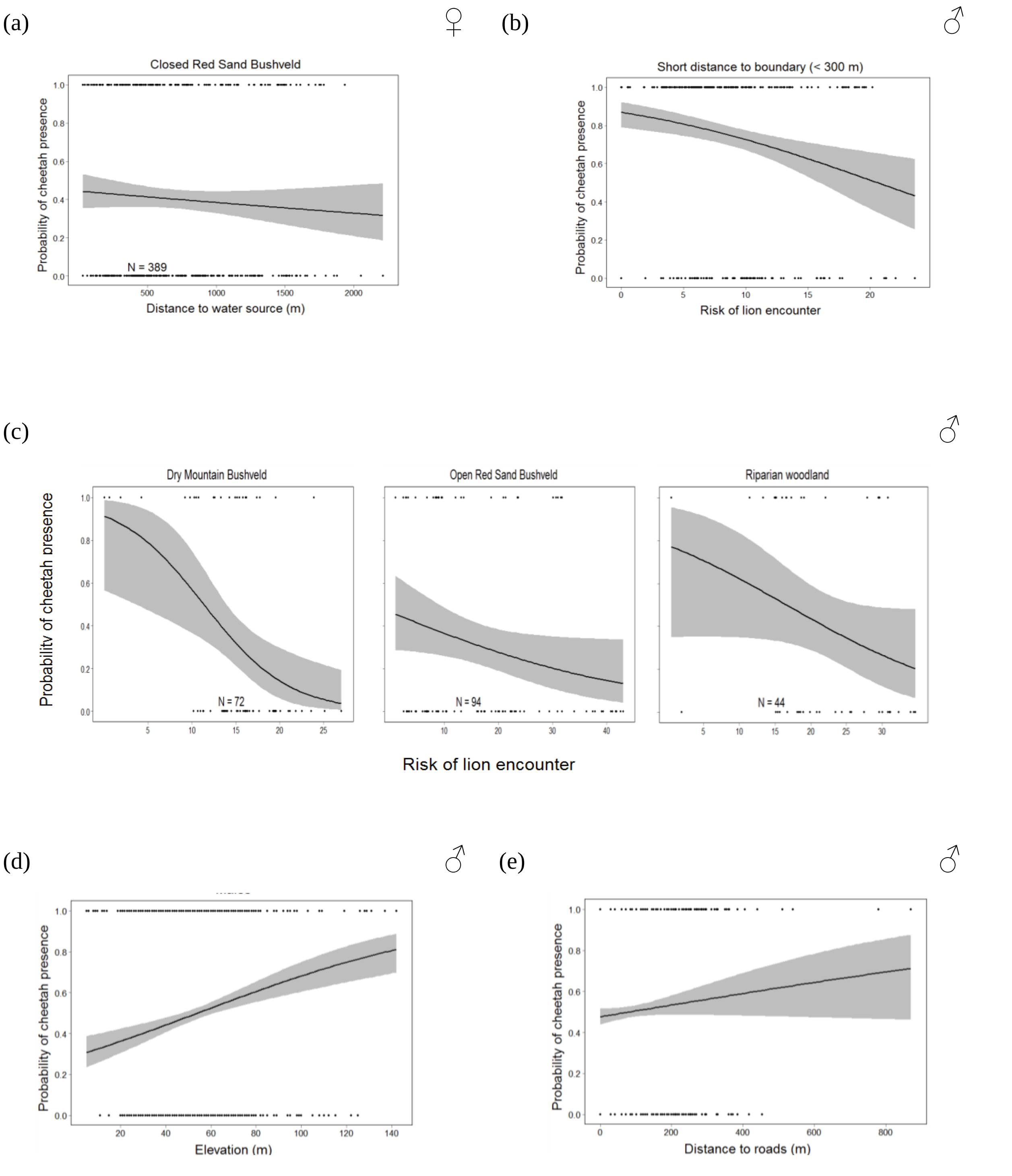

Figure S1 Relationship between the presence of: (a) female cheetahs and the distance to water sources in closed red sand bushveld; (b) cheetah male coalitions and the risk of encountering a lion in close distance to the boundary; (c) cheetah male coalitions and the risk of encountering a lion in different habitat types; (d) cheetah male coalitions and elevation; and (e) cheetah male coalitions and distance to roads.
