# Supplementary material for: To Kill, Stay or Flee: The Effects of Lions and Landscape Factors on Habitat and Kill Site Selection of Cheetahs in South Africa
Source: PLoS One. 2015 Feb 18;10(2):e0117743. doi: 10.1371/journal.pone.0117743 (PMC4333767; doi:10.1371/journal.pone.0117743)
Supplement: S2 Fig — (PDF) [file pone.0117743.s002.pdf]

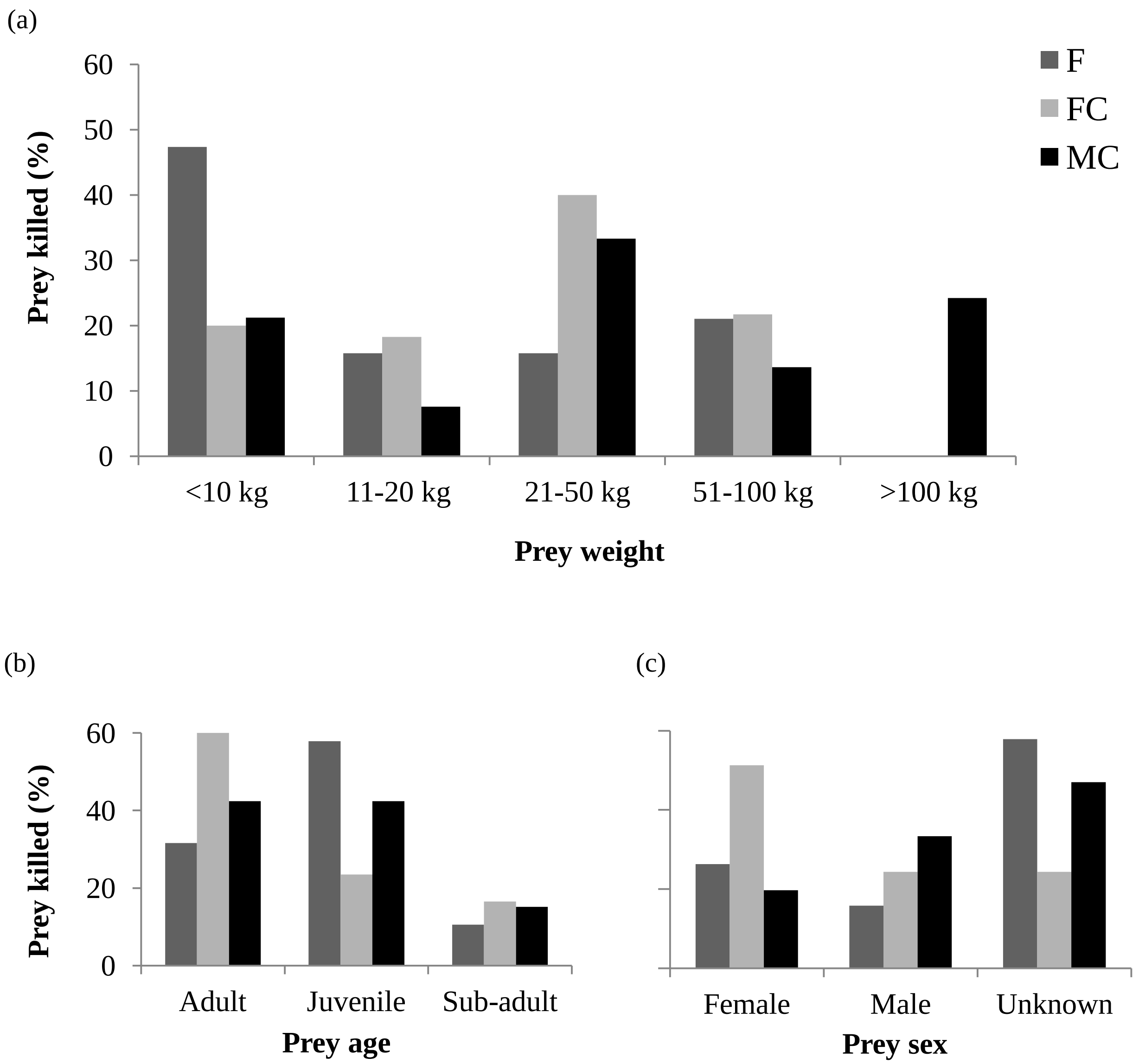

Figure S2 Prey categories by: (a) weight, (b) age, and (c) sex of kills (*n* = 200) made by cheetahs from three types of social groups (F = solitary females, FC = females with cubs, MC = male coalitions).
