# Supplementary material for: To Kill, Stay or Flee: The Effects of Lions and Landscape Factors on Habitat and Kill Site Selection of Cheetahs in South Africa
Source: PLoS One. 2015 Feb 18;10(2):e0117743. doi: 10.1371/journal.pone.0117743 (PMC4333767; doi:10.1371/journal.pone.0117743)
Supplement: S3 Fig — (PDF) [file pone.0117743.s003.pdf]

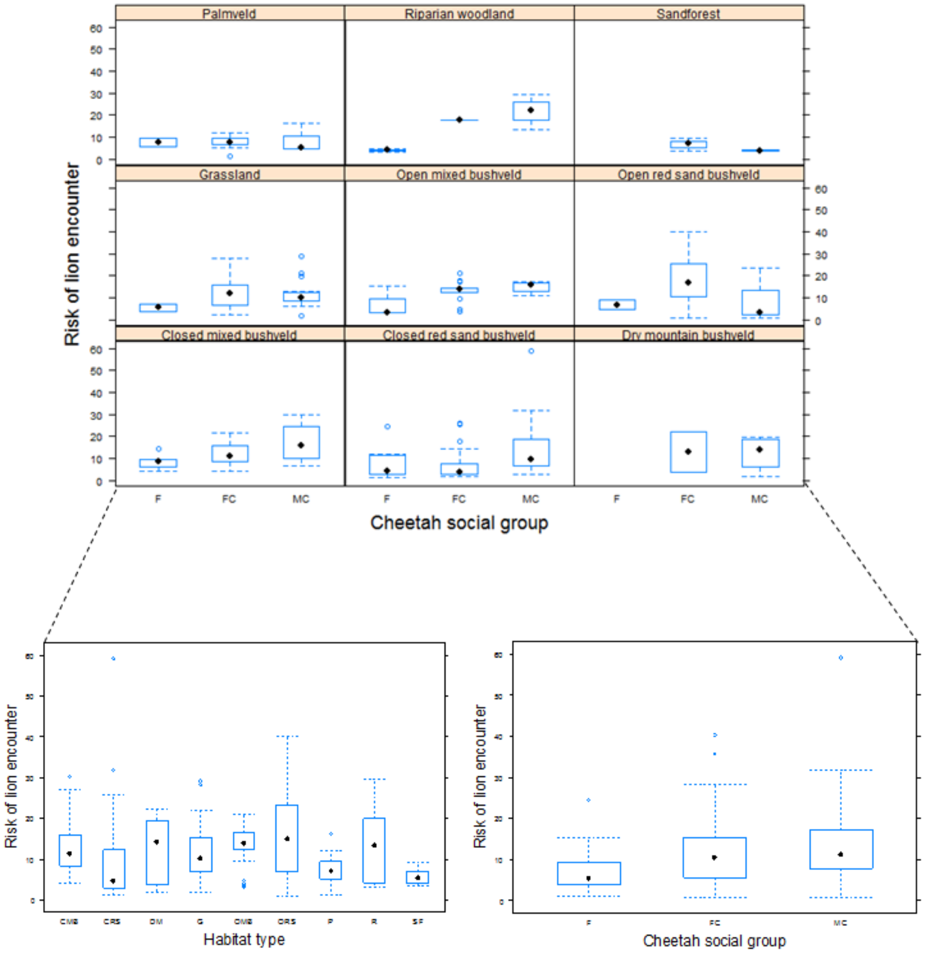

Figure S3 Risk of lion encounter at kill sites among habitat types and cheetah social groups (F = single females, FC = females with cubs, MC = male coalitions).
