# Supplementary material for: To Kill, Stay or Flee: The Effects of Lions and Landscape Factors on Habitat and Kill Site Selection of Cheetahs in South Africa
Source: PLoS One. 2015 Feb 18;10(2):e0117743. doi: 10.1371/journal.pone.0117743 (PMC4333767; doi:10.1371/journal.pone.0117743)
Supplement: S1 Table — (DOCX) [file pone.0117743.s005.docx]

**Table S1** **Third-order habitat selection (locations vs. randomly sampled locations) of female cheetahs (*n* = 4), showing multi-model (Generalized Linear Mixed Models) beta coefficient averages of parameters (within the intercept are included closed mixed bushveld and random locations).**

| **Parameter** | **Estimate^+^** | **Std. Error** | **z value** | **Pr(>\|z\|)** | | **Relative importance^†^** |
| --- | --- | --- | --- | --- | --- | --- |
| (Intercept) | -0.210786 | 0.100309 | 2.101 | 0.0356 | * |  |
| Closed Red Sand Bushveld (CRS) | -0.128127 | 0.151551 | 0.845 | 0.3979 |  | 1.00 |
| Dry Mountain Bushveld (DM) | 1.372295 | 0.601413 | 2.282 | 0.0225 | * | 1.00 |
| Grassland (G) | 0.622487 | 0.146129 | 4.26 | 2.05E-05 | *** | 1.00 |
| Open Mixed Bushveld (OMB) | 0.313763 | 0.154164 | 2.035 | 0.0418 | * | 1.00 |
| Open Red Sand Bushveld (ORS) | -0.296779 | 0.212497 | 1.397 | 0.1625 |  | 1.00 |
| Palmveld (P) | 0.893905 | 0.205337 | 4.353 | 1.34E-05 | *** | 1.00 |
| Riparian woodland (R) | 0.945225 | 0.471646 | 2.004 | 0.0451 | * | 1.00 |
| Sand Forest (SF) | 0.060913 | 0.301452 | 0.202 | 0.8399 |  | 1.00 |
| Boundary (B) | -0.519986 | 0.102849 | 5.056 | 4.00E-07 | *** | 1.00 |
| Water bodies (WB) | 0.411818 | 0.265176 | 1.553 | 0.1204 |  | 1.00 |
| CRS x WB | -0.662942 | 0.325403 | 2.037 | 0.0416 | * | 0.98 |
| DM x WB | -0.169981 | 1.348366 | 0.126 | 0.8997 |  | 0.98 |
| G x WB | 0.253951 | 0.345478 | 0.735 | 0.4623 |  | 0.98 |
| OMB x WB | -0.752273 | 0.44641 | 1.685 | 0.092 | **.** | 0.98 |
| ORS x WB | 0.136788 | 0.348126 | 0.393 | 0.6944 |  | 0.98 |
| P x WB | 0.640767 | 0.538594 | 1.19 | 0.2342 |  | 0.98 |
| R x WB | 0.721185 | 0.886733 | 0.813 | 0.416 |  | 0.98 |
| SF x WB | -1.634533 | 0.862675 | 1.895 | 0.0581 | **.** | 0.98 |
| Lion risk (LR) | -0.075021 | 0.110277 | 0.68 | 0.4963 |  | 0.57 |
| Elevation (E) | -0.037486 | 0.11839 | 0.317 | 0.7515 |  | 0.33 |
| Roads (Ro) | -0.003991 | 0.108213 | 0.037 | 0.9706 |  | 0.30 |
| B x LR | 0.190443 | 0.255303 | 0.746 | 0.4557 |  | 0.18 |
| LR x WB | -0.129271 | 0.184349 | 0.701 | 0.4832 |  | 0.18 |
| LR x E | 0.166298 | 0.268374 | 0.62 | 0.5355 |  | 0.06 |
| LR x Ro | 0.082803 | 0.262472 | 0.315 | 0.7524 |  | 0.05 |
| CRS x LR | -0.111599 | 0.318033 | 0.351 | 0.7257 |  | 0.01 |
| DM x LR | -1.754833 | 1.621009 | 1.083 | 0.279 |  | 0.01 |
| G x LR | 0.360963 | 0.359272 | 1.005 | 0.315 |  | 0.01 |
| OMB x LR | -0.441423 | 0.350544 | 1.259 | 0.2079 |  | 0.01 |
| ORS x LR | -0.141286 | 0.3422 | 0.413 | 0.6797 |  | 0.01 |
| P x LR | 0.22103 | 0.681594 | 0.324 | 0.7457 |  | 0.01 |
| R x LR | 0.785887 | 0.650997 | 1.207 | 0.2274 |  | 0.01 |
| SF x LR | 0.1619 | 0.503004 | 0.322 | 0.7476 |  | 0.01 |

‘**.**’ *P* < 0.1, ‘*’ *P* < 0.05, ‘**’ *P* < 0.01, ‘***’ for *P* < 0.001.

**^+^** Effect sizes have been scaled.

^†^ Sum of the *Akaike weights* over all of the models in which the parameter of interest appears.
