# Supplementary material for: To Kill, Stay or Flee: The Effects of Lions and Landscape Factors on Habitat and Kill Site Selection of Cheetahs in South Africa
Source: PLoS One. 2015 Feb 18;10(2):e0117743. doi: 10.1371/journal.pone.0117743 (PMC4333767; doi:10.1371/journal.pone.0117743)
Supplement: S2 Table — (DOCX) [file pone.0117743.s006.docx]

**Table S2 Third-order habitat selection (locations vs. randomly sampled locations) of male cheetah coalitions (*n* = 2), showing multi-model (Generalized Linear Mixed Models) beta coefficient averages of parameters (within the intercept are included closed mixed bushveld and random locations).**

| **Parameter** | **Estimate^+^** | **Std. Error** | **z value** | **Pr(>\|z\|)** | | **Relative importance^†^** |
| --- | --- | --- | --- | --- | --- | --- |
| (Intercept) | -0.18406 | 0.14669 | 1.255 | 0.209555 |  |  |
| Closed Red Sand Bushveld (CRS) | -0.13461 | 0.25493 | 0.528 | 0.597493 |  | 1.00 |
| Dry Mountain Bushveld (DM) | -1.32398 | 0.36091 | 3.668 | 0.000244 | *** | 1.00 |
| Grassland (G) | 0.77797 | 0.1987 | 3.915 | 9.03E-05 | *** | 1.00 |
| Open Mixed Bushveld (OMB) | 0.30941 | 0.19592 | 1.579 | 0.114268 |  | 1.00 |
| Open Red Sand Bushveld (ORS) | -0.52105 | 0.29964 | 1.739 | 0.082045 | **.** | 1.00 |
| Palmveld (P) | -0.11997 | 0.39936 | 0.3 | 0.763866 |  | 1.00 |
| Riparian woodland (R) | 0.68403 | 0.50532 | 1.354 | 0.175842 |  | 1.00 |
| Sand Forest (SF) | 0.15336 | 0.58561 | 0.262 | 0.793416 |  | 1.00 |
| Boundary (B) | -0.66479 | 0.15798 | 4.208 | 2.58E-05 | *** | 1.00 |
| Elevation (E) | 0.68198 | 0.16454 | 4.145 | 3.40E-05 | *** | 1.00 |
| Lion risk (LR) | -0.11127 | 0.32187 | 0.346 | 0.729578 |  | 0.99 |
| Roads (Ro) | 0.24302 | 0.1269 | 1.915 | 0.055493 | **.** | 0.81 |
| B x LR | 0.87382 | 0.38124 | 2.292 | 0.021902 | * | 0.82 |
| Water bodies (WB) | 0.02827 | 0.22468 | 0.126 | 0.899866 |  | 0.53 |
| LR x WB | -0.43522 | 0.25809 | 1.686 | 0.091733 | **.** | 0.33 |
| LR x Ro | -0.19722 | 0.23781 | 0.829 | 0.406921 |  | 0.27 |
| LR x E | -0.15208 | 0.28448 | 0.535 | 0.592933 |  | 0.29 |
| CRS x LR | -0.34469 | 0.49602 | 0.695 | 0.487108 |  | 0.23 |
| DM x LR | -2.61698 | 1.20653 | 2.169 | 0.030082 | * | 0.23 |
| G x LR | -0.68339 | 0.45019 | 1.518 | 0.129011 |  | 0.23 |
| OMB x LR | -0.45803 | 0.63453 | 0.722 | 0.470394 |  | 0.23 |
| ORS x LR | -1.21447 | 0.61043 | 1.99 | 0.04664 | * | 0.23 |
| P x LR | 0.29632 | 0.96372 | 0.307 | 0.758483 |  | 0.23 |
| R x LR | -1.70743 | 0.81092 | 2.106 | 0.035243 | * | 0.23 |
| SF x LR | -0.16686 | 0.65117 | 0.256 | 0.797761 |  | 0.23 |
| CRS x WB | -0.37336 | 0.57802 | 0.646 | 0.518324 |  | 0.11 |
| DM x WB | -0.30353 | 1.22255 | 0.248 | 0.803919 |  | 0.11 |
| G x WB | 0.60026 | 0.47655 | 1.26 | 0.207821 |  | 0.11 |
| OMB x WB | -0.30064 | 0.4963 | 0.606 | 0.544667 |  | 0.11 |
| ORS x WB | 1.50883 | 0.86462 | 1.745 | 0.080972 | **.** | 0.11 |
| P x WB | -0.03888 | 0.6602 | 0.059 | 0.953033 |  | 0.11 |
| R x WB | -0.76032 | 0.75967 | 1.001 | 0.316899 |  | 0.11 |
| SF x WB | -0.60418 | 2.39234 | 0.253 | 0.800617 |  | 0.11 |

‘**.**’ *P* < 0.1, ‘*’ *P* < 0.05, ‘**’ *P* < 0.01, ‘***’ for *P* < 0.001.

**^+^** Effect sizes have been scaled.

^†^ Sum of the *Akaike weights* over all of the models in which the parameter of interest appears.
