# Supplementary material for: To Kill, Stay or Flee: The Effects of Lions and Landscape Factors on Habitat and Kill Site Selection of Cheetahs in South Africa
Source: PLoS One. 2015 Feb 18;10(2):e0117743. doi: 10.1371/journal.pone.0117743 (PMC4333767; doi:10.1371/journal.pone.0117743)
Supplement: S3 Table — (DOCX) [file pone.0117743.s007.docx]

**Table S3** **Fourth-order habitat selection (kill sites vs. locations) of cheetahs (*n* = 6) in winter, showing multi-model (Generalized Linear Mixed Models) beta coefficient averages of parameters (within the intercept are included closed mixed bushveld, solitary female cheetahs and random locations).**

| **Parameter** | **Estimate^+^** | **Std. Error** | **z value** | **Pr(>\|z\|)** | | **Relative importance^†^** |
| --- | --- | --- | --- | --- | --- | --- |
| (Intercept) | -2.57379 | 0.42443 | 6.064 | <2e-16 | *** |  |
| Females with cubs (FC) | 0.68924 | 0.33573 | 2.053 | 0.0401 | * | 0.58 |
| Male coalitions (MC) | 0.72138 | 0.43062 | 1.675 | 0.0939 | **.** | 0.58 |
| Closed Red Sand Bushveld (CRS) | 0.36808 | 0.37556 | 0.98 | 0.327 |  | 0.97 |
| Dry Mountain Bushveld (DM) | 0.31586 | 0.63519 | 0.497 | 0.619 |  | 0.97 |
| Grassland (G) | -0.40048 | 0.36954 | 1.084 | 0.2785 |  | 0.97 |
| Open Mixed Bushveld (OMB) | 0.04624 | 0.42421 | 0.109 | 0.9132 |  | 0.97 |
| Open Red Sand Bushveld (ORS) | 0.31642 | 0.44967 | 0.704 | 0.4816 |  | 0.97 |
| Palmveld (P) | -1.20687 | 0.55141 | 2.189 | 0.0286 | * | 0.97 |
| Riparian woodland (R) | 0.94336 | 0.57593 | 1.638 | 0.1014 |  | 0.97 |
| Sand Forest (SF) | 0.92976 | 0.56924 | 1.633 | 0.1024 |  | 0.97 |
| Roads (Ro) | 0.19836 | 0.10608 | 1.87 | 0.0615 | **.** | 0.80 |
| Boundary (B) | -0.14921 | 0.12385 | 1.205 | 0.2283 |  | 0.44 |
| Elevation (E) | -0.09365 | 0.13457 | 0.696 | 0.4865 |  | 0.44 |
| Water bodies (WB) | -0.09656 | 0.11339 | 0.852 | 0.3945 |  | 0.39 |
| Lion risk (LR) | -0.01386 | 0.22661 | 0.061 | 0.9512 |  | 0.51 |
| LR x E | 0.17422 | 0.12607 | 1.382 | 0.167 |  | 0.13 |
| LR x Ro | -0.06475 | 0.13301 | 0.487 | 0.6264 |  | 0.12 |
| FC x LR | 0.49763 | 0.34928 | 1.425 | 0.1542 |  | 0.09 |
| MC x LR | 0.31214 | 0.35171 | 0.887 | 0.3748 |  | 0.09 |
| LR x WB | -0.06031 | 0.10407 | 0.58 | 0.5622 |  | 0.07 |
| CRS x LR | 0.67522 | 0.37592 | 1.796 | 0.0725 | **.** | 0.01 |
| DM x LR | 0.44353 | 0.81755 | 0.543 | 0.5875 |  | 0.01 |
| G x LR | 0.4005 | 0.42689 | 0.938 | 0.3482 |  | 0.01 |
| OMB x LR | -0.02753 | 0.55467 | 0.05 | 0.9604 |  | 0.01 |
| ORS x LR | 0.53701 | 0.40787 | 1.317 | 0.188 |  | 0.01 |
| P x LR | 0.55218 | 1.02371 | 0.539 | 0.5896 |  | 0.01 |
| R x LR | 0.53533 | 0.60386 | 0.887 | 0.3753 |  | 0.01 |
| SF x LR | -1.27097 | 1.76422 | 0.72 | 0.4713 |  | 0.01 |
| CRS x WB | -0.32097 | 0.45548 | 0.705 | 0.481 |  | 0.00 |
| DM x WB | -0.31473 | 0.82274 | 0.383 | 0.7021 |  | 0.00 |
| G x WB | -0.03768 | 0.44691 | 0.084 | 0.9328 |  | 0.00 |
| OMB x WB | -0.57867 | 0.61312 | 0.944 | 0.3453 |  | 0.00 |
| ORS x WB | 0.05006 | 0.44502 | 0.112 | 0.9104 |  | 0.00 |
| P x WB | -0.72463 | 0.81181 | 0.893 | 0.3721 |  | 0.00 |
| R x WB | 0.18221 | 0.59131 | 0.308 | 0.758 |  | 0.00 |
| SF x WB | 0.3069 | 0.84802 | 0.362 | 0.7174 |  | 0.00 |

‘**.**’ *P* < 0.1, ‘*’ *P* < 0.05, ‘**’ *P* < 0.01, ‘***’ for *P* < 0.001.

**^+^** Effect sizes have been scaled.

^†^ Sum of the *Akaike weights* over all of the models in which the parameter of interest appears.
