# Supplementary material for: To Kill, Stay or Flee: The Effects of Lions and Landscape Factors on Habitat and Kill Site Selection of Cheetahs in South Africa
Source: PLoS One. 2015 Feb 18;10(2):e0117743. doi: 10.1371/journal.pone.0117743 (PMC4333767; doi:10.1371/journal.pone.0117743)
Supplement: S4 Table — (DOCX) [file pone.0117743.s008.docx]

**Table S4** **Fourth-order habitat selection (kill sites vs. locations) of cheetahs (*n* = 6) in summer, showing multi-model (Generalized Linear Mixed Models) beta coefficient averages of parameters (within the intercept are included closed mixed bushveld, solitary female cheetahs and random locations).**

| **Parameter** | **Estimate^+^** | **Std. Error** | **z value** | **Pr(>\|z\|)** | | **Relative importance^†^** |
| --- | --- | --- | --- | --- | --- | --- |
| (Intercept) | -1.90E+00 | 4.10E-01 | 4.633 | 3.60E-06 | *** |  |
| Closed Red Sand Bushveld (CRS) | 9.17E-01 | 4.16E-01 | 2.202 | 0.0277 | * | 0.58 |
| Dry Mountain Bushveld (DM) | 5.01E-01 | 1.35E+00 | 0.372 | 0.7097 |  | 0.58 |
| Grassland (G) | -3.90E-02 | 3.45E-01 | 0.113 | 0.91 |  | 0.58 |
| Open Mixed Bushveld (OMB) | -6.73E-01 | 3.84E-01 | 1.753 | 0.0797 | **.** | 0.58 |
| Open Red Sand Bushveld (ORS) | 4.02E-01 | 6.46E-01 | 0.622 | 0.5337 |  | 0.58 |
| Palmveld (P) | -1.61E-01 | 8.25E-01 | 0.195 | 0.8455 |  | 0.58 |
| Riparian woodland (R) | -1.32E+00 | 1.49E+00 | 0.89 | 0.3734 |  | 0.58 |
| Sand Forest (SF) | 9.22E-01 | 1.03E+03 | 0.001 | 0.9993 |  | 0.58 |
| Boundary (B) | 3.12E-01 | 1.30E-01 | 2.394 | 0.0167 | * | 0.87 |
| Water bodies (WB) | 1.68E-01 | 3.36E-01 | 0.498 | 0.6182 |  | 0.55 |
| CRS x WB | -3.17E-02 | 4.13E-01 | 0.077 | 0.9387 |  | 0.29 |
| DM x WB | 6.65E-01 | 1.42E+00 | 0.467 | 0.6405 |  | 0.29 |
| G x WB | -9.86E-01 | 3.82E-01 | 2.579 | 0.0099 | ** | 0.29 |
| OMB x WB | -1.37E+00 | 5.40E-01 | 2.541 | 0.011 | * | 0.29 |
| ORS x WB | -2.37E-01 | 3.87E-01 | 0.612 | 0.5403 |  | 0.29 |
| P x WB | -1.65E+00 | 8.49E-01 | 1.947 | 0.0516 | **.** | 0.29 |
| R x WB | -1.19E+00 | 1.23E+00 | 0.966 | 0.3339 |  | 0.29 |
| SF x WB | -6.85E-01 | 9.12E+02 | 0.001 | 0.9994 |  | 0.29 |
| Females with cubs (FC) | 1.13E-01 | 4.74E-01 | 0.238 | 0.8118 |  | 0.46 |
| Male coalitions (MC) | -5.66E-01 | 5.52E-01 | 1.026 | 0.3048 |  | 0.46 |
| Elevation (E) | 2.00E-01 | 1.69E-01 | 1.187 | 0.2353 |  | 0.52 |
| Lion risk (LR) | -1.33E-01 | 2.14E-01 | 0.622 | 0.5341 |  | 0.47 |
| Roads (Ro) | -1.21E-02 | 1.17E-01 | 0.103 | 0.9181 |  | 0.30 |
| LR x E | 6.31E-02 | 1.34E-01 | 0.472 | 0.6372 |  | 0.08 |
| LR x WB | 3.77E-02 | 1.15E-01 | 0.327 | 0.7437 |  | 0.07 |
| LR x Ro | -5.98E-02 | 1.19E-01 | 0.502 | 0.6158 |  | 0.05 |
| FC x LR | 2.49E-01 | 6.35E-01 | 0.392 | 0.695 |  | 0.03 |
| MC x LR | 1.66E-01 | 6.54E-01 | 0.253 | 0.7999 |  | 0.03 |
| CRS x LR | -1.22E-02 | 5.05E-01 | 0.024 | 0.9808 |  | 0.01 |
| DM x LR | -2.38E-01 | 8.59E-01 | 0.277 | 0.7815 |  | 0.01 |
| G x LR | 4.13E-02 | 4.20E-01 | 0.098 | 0.9216 |  | 0.01 |
| OMB x LR | 5.77E-01 | 4.34E-01 | 1.33 | 0.1836 |  | 0.01 |
| ORS x LR | 3.95E-01 | 5.15E-01 | 0.768 | 0.4425 |  | 0.01 |
| P x LR | -7.52E-01 | 1.07E+00 | 0.701 | 0.4833 |  | 0.01 |
| R x LR | -2.21E+00 | 3.34E+00 | 0.661 | 0.5084 |  | 0.01 |
| SF x LR | -1.54E+02 | 1.46E+04 | 0.011 | 0.9916 |  | 0.01 |

‘.’ *P* < 0.1, ‘*’ *P* < 0.05, ‘**’ *P* < 0.01, ‘***’ for *P* < 0.001.

**^+^** Effect sizes have been scaled.

^†^ Sum of the *Akaike weights* over all of the models in which the parameter of interest appears.
